# Controversy: in heart failure patients with a reduced ejection fraction and left bundle branch block, conduction system pacing can be a valid alternative to biventricular pacing—pro and contra

**DOI:** 10.1093/europace/euaf312

**Published:** 2025-12-23

**Authors:** Haran Burri, Christophe Leclercq, Nathalie Behar, Jean-Claude Deharo, Marek Jastrzebski, Jacqueline Joza, Jens Cosedis Nielsen

**Affiliations:** Cardiac Pacing Unit, Cardiology Department, University Hospital of Geneva, Rue Gabrielle Perret Gentil 4, 1211 Geneva 14, Switzerland; Rennes University Hospital, CIC-IT 804; LTSI INSERM 1099. Rennes, France; Rennes University Hospital, CIC-IT 804; LTSI INSERM 1099. Rennes, France; Department of Cardiology, L'hôpital de la Timone, Marseille 13005, France; First Department of Cardiology, Interventional Electrocardiology and Hypertension, Jagiellonian University, Medical College, Krakow, Poland; Department of Medicine, McGill University Health Centre, 1001 Decarie Boulevard, Montréal, Québec, Canada, H4A 3J1; Department of Cardiology, Aarhus University Hospital, Palle Juul-Jensens Blv 99, 8200 Aarhus N, Denmark

**Keywords:** Cardiac resynchronization therapy, Conduction system pacing, Biventricular pacing, Heart failure

## Abstract

For patients with heart failure (HF) and bundle branch block, cardiac resynchronization therapy (CRT) by biventricular pacing (BiVP) has been found effective and has been widely used for around 20 years. The effects of BiVP are well documented in a row of large randomized controlled trials (RCTs) with long-term follow-up to include prolonged survival, less HF hospitalizations, and better quality of life for the patients. More recently, conduction system pacing (CSP) as His bundle pacing or left bundle branch area pacing has been introduced for CRT and shown to in best cases establish a normal or near-to-normal electrical activation of the left ventricular myocardium. Data from large RCTs documenting the beneficial effects of CSP are awaited. Currently, the question is to what extent the contemporary literature supports a transition from BiVP to CSP for CRT in patients with HF and bundle branch block. This *Europace* Controversy article presents opposing viewpoints on this topic. H.B., M.J., and J.J. argue in favour of CSP being superior to BiVP. Conversely, C.L., N.B., and J.C.D. advocate for BiVP still being the first choice for CRT. This Controversy aims to present data and their interpretation from different expert perspectives on an important topic in CRT for HF.

## Graphical Abstract

**Graphical Abstract:**
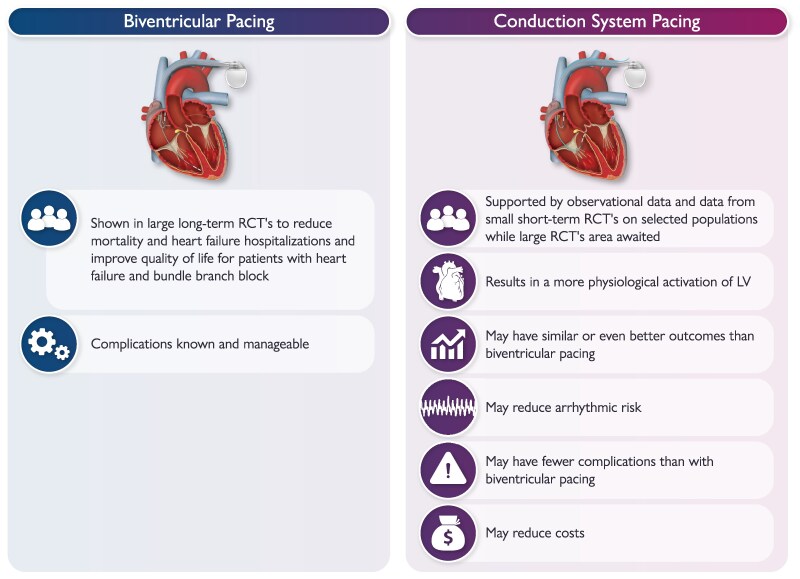

## Figures and Tables

**Figure 1:**
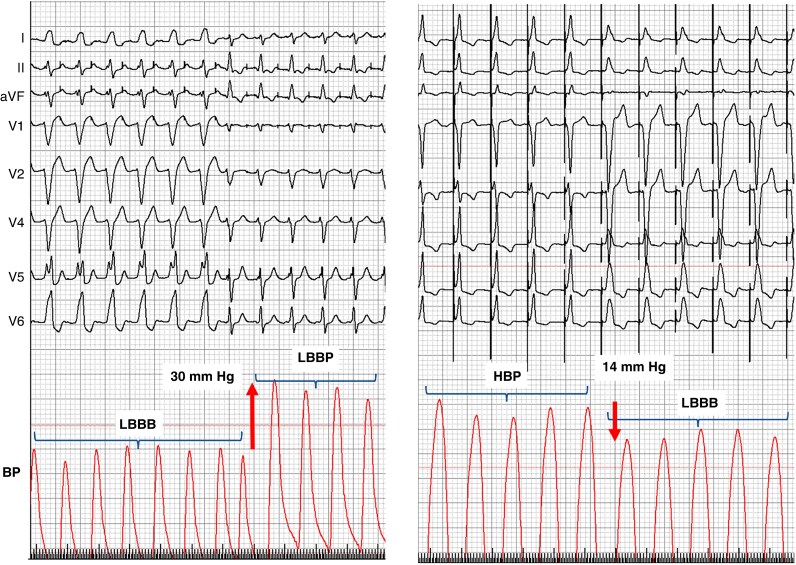
An on-off phenomenon: correction of LBBB-induced dyssynchrony by LBBP (left panel) or HBP (right panel) resulted in an immediate, beat-to-beat increase in systolic blood pressure, as measured in the femoral artery. The spectacular shortening of ventricular activation time, as evidenced by shortening of the V6 R-wave peak time, and the improvement in the mechanical performance of the heart offer undeniable evidence of the acute resynchronization effect of CSP. CSP, conduction system pacing; HBP, His bundle pacing; LBBB, left bundle branch block; LBBP, left bundle branch pacing.

**Figure 2:**
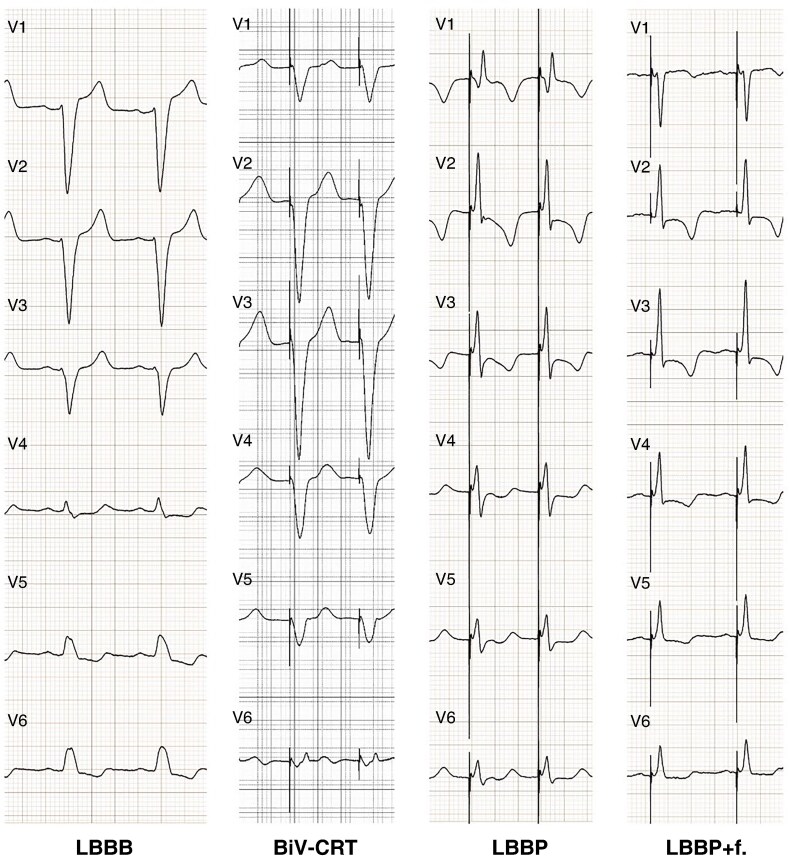
In contrast to LBBP, BiVP-CRT did not narrow the baseline QRS in a patient with LBBB. Adding fusion with right bundle branch block activation to LBBP (LBBP+f.) resulted in further QRS narrowing and near-complete restoration of physiological activation of the heart. BiVP-CRT, biventricular pacing-based cardiac resynchronization therapy; LBBB, left bundle branch block; LBBP, left bundle branch pacing.

**Figure 3:**
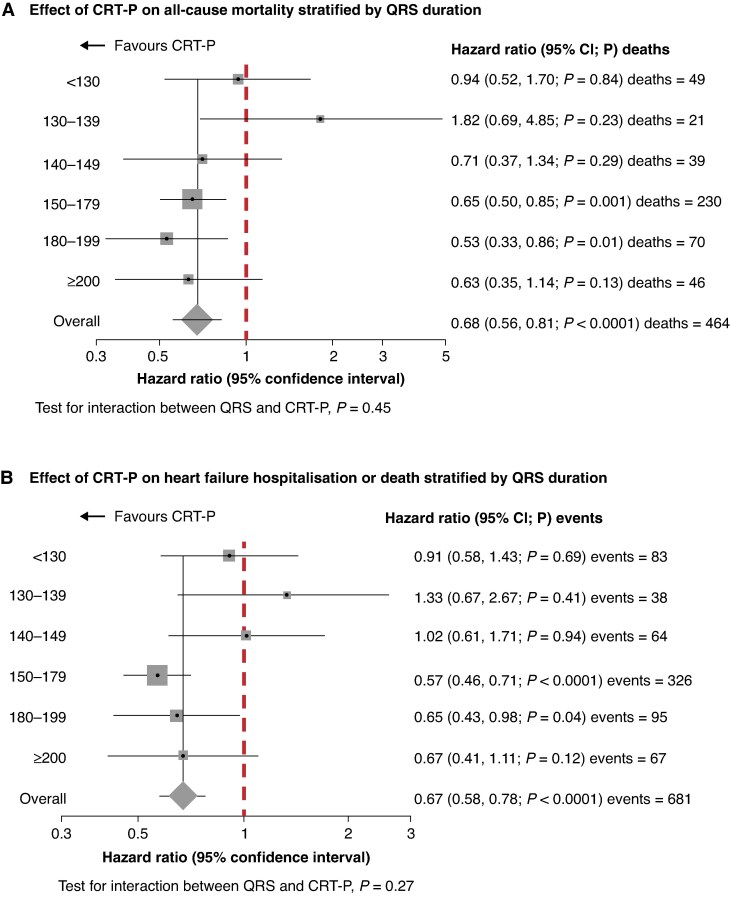
(*A*) Effect of CRT-P on all-cause mortality stratified by QRS duration. Test for heterogeneity, *P* = 0.104. (*B*) Effect of CRT-P on HF hospitalization or death stratified by QRS duration. Test for heterogeneity, *P* = 0.269. CI, confidence interval; CRT-P, cardiac resynchronization therapy pacemaker; HF, heart failure. Reproduced from Cleland *et al.*^3^

**Table 1:** Comparison of CRT and CSP

Parameter	BiVP	CSP
Mechanism	Direct biventricular stimulation	Recruitment of intrinsic conduction
Evidence base	Strong RCT data	Emerging limited RCTs, mainly observational data from experienced centres
Implantation process	Standardized, reproducible	Can be technically demanding
Applicability	Broad (even with distal block)	May be limited by septal fibrosis and/or compromised distal conduction
Threshold stability	Usually stable	May be variable with HBP, usually stable for LBBAP
Technology	Mature/fully supported by PPM and ICD platforms	Still evolving (lead technology, device compatibility)

BiVP, biventricular pacing; CRT, cardiac resynchronization therapy; CSP, conduction system pacing; HBP, His bundle pacing; ICD, implantable cardioverter defibrillator; LBBAP, left bundle branch area pacing; PPM, permanent pacemaker; RCT, randomized controlled trial.

## Introduction

https://orcid.org/0000-0003-0631-0010NielsenJens Cosedis

In patients with heart failure (HF) with reduced ejection fraction (HFrEF), pharmacological treatment with four guideline-recommended drug classes leads to improved pump function of the heart as well as better clinical outcomes including functional capacity, quality of life, and freedom from hospitalization and death.^1^ For the subgroup of patients with HF and reduced ejection fraction who also have prolonged ventricular activation as expressed in QRS widening in the ECG, cardiac resynchronization therapy (CRT) may be relevant.^1,2^

Cardiac resynchronization therapy appeared first in the literature in case series in the mid-1990s^3,4^ followed by smaller studies and the first larger randomized controlled trial (RCT) published as the MIRACLE study in 2002.^5^ Since then and until few years ago, CRT has been synonymous with biventricular pacing (BiVP) from both the right ventricle (RV) and the epicardial part of the left ventricle (LV) via a coronary sinus branch with biventricular stimulation or left-ventricular-only stimulation.

After publication of the two large double-blind RCTs, the CARE-HF trial^6^ and the COMPANION trial,^7^ both with moderate- to long-term follow-up and a composite of death and hospitalization as primary endpoint, CRT entered the European Society of Cardiology (ESC) guidelines, first in 2007 in the guideline of cardiac pacing and CRT^8^ and the following year in the 2008 version of the ESC guideline on the treatment of HF.^9^ Initially, CRT was indicated for patients with New York Heart Association (NYHA) class III-IV, LV dilatation, LV ejection fraction (LVEF) ≤ 35%, and QRS width 120 ms or wider. In a 2010 update of the guidelines,^10^ patients with NYHA class II were included in the recommendations, and the requirement for LV dilatation disappeared. In the most recent guidelines on cardiac pacing and CRT as well as on the treatment for HF, both published in 2021,^1,2^ CRT still is indicated as BiVP in symptomatic HF patients with LVEF ≤ 35% despite optimal medical treatment, QRS width 130 ms or wider, and with the strongest recommendations in those with sinus rhythm, left bundle branch block (LBBB) QRS morphology, and QRS width 150 ms or wider. Only in cases where a LV lead cannot be placed via the coronary sinus, and thus BiVP not established, a recommendation to use His bundle pacing (HBP) was adopted.

It is well documented that CRT as BiVP prolongs life, reduces HF hospitalization, and improves functional capacity and quality of life for this patient cohort.

Within the last 5–8 years, more attention has been given to a potential alternative to BiVP in establishing CRT, namely conduction system pacing (CSP). It has been known since almost 50 years that in bundle branch block, functional longitudinal dissociation in the His bundle may allow that pacing distal to such areas or lesions may enable normalization of the bundle branch block and conduction with a normal, narrow QRS complex in some cases.^11,12^ Pacing the conduction system can be done as HBP or as left bundle branch pacing (LBBP), the latter described first in a patient with dilated cardiomyopathy and LBBB in a case report in 2017.^13^ Even as early as 2003, LV septal pacing was found to maintain normal LV pump function in an acute canine model.^14^ So far, observational data indicate that CSP may be an alternative to BiVP for CRT in patients with HF and bundle branch block, and similar findings have resulted from small short-term randomized trials on highly selected patients. However, to enter upcoming ESC guidelines on cardiac pacing and CRT, CSP must be found superior or at least non-inferior to BiVP in sufficiently large and well-powered RCTs with a clinically relevant follow-up for treatment with cardiac implantable electronic devices.^15^

Currently, the question is to what extent the contemporary literature supports a transition from BiVP to CSP for CRT in patients with HF and bundle branch block. This topic is further discussed and debated in a pro-and-con format below.

### Data availability

No original data presented, and thus not relevant.

## Pro

https://orcid.org/0000-0002-4393-5338BurriHaranhttps://orcid.org/0000-0002-3318-6601JastrzebskiMarekhttps://orcid.org/0000-0003-0956-688XJozaJacqueline

There is no doubt that BiVP has proved to be effective in patients with LBBB and HFrEF. Nevertheless, there remain several shortcomings including technical limitations and a relatively high non-responder rate, with unresolved questions regarding patient selection, acute procedural endpoints for confirmation of resynchronization, and optimization of programming. These challenges have persisted despite more than a quarter century of therapeutic evolution, and they are unlikely to ever be fully resolved.

Over the last years, CSP has been gaining momentum as an alternative to BiVP.^1–3^ Initially, this was primarily driven by the physiological imperative and the personal experience of early adopters, based on their anecdotal yet undeniable evidence that CSP resulted in immediate and impressive electrical and haemodynamic resynchronization and offered an excellent bailout approach for failed BiVP (*Figures 1* and *2*). Growing evidence of safety and efficacy has positioned CSP in practice guidelines and consensus documents.^4,5^

We hereby present arguments which support the more contemporary form of CRT, which also includes CSP.

### Conduction system pacing is more physiological than biventricular pacing

His bundle pacing can correct intraventricular activation in roughly 60% of patients with a LBBB pattern.^6^ However, challenges related to lead placement and the high pacing outputs often required to correct LBBB—particularly in cases involving distal intra-Hisian block—limit the practical use of HBP as a form of CRT. Left bundle branch area pacing (LBBAP), by targeting a more distal pacing site within the conduction system over a broad area,^7–9^ overcomes several of these limitations and broadens the use of CSP for CRT. By harnessing the patient’s native conduction system network, CSP results in near-synchronous ventricular electrical activation compared to BiVP, which only activates the ventricles from two (or three in case of multipoint pacing)^10,11^ distinct myocardial foci. Furthermore, during CSP, electrical activation follows the natural course of endo-epicardial electrical activation, whereas pacing from the coronary sinus follows an epi-endocardial sequence. As a result, LBBAP translates into substantially greater QRS narrowing from baseline (*Figure 2*)—a recognized marker of acute electrical resynchronization that has been associated with improved survival in patients treated with BiVP.^12^ Moreover, maintaining the physiological contour of the paced QRS complex makes it feasible to gauge intraprocedurally the degree of restoration of physiological activation (e.g. by evaluating the V6 R-wave peak time).^13–15^ Computer modelling has shown shorter LV activation time (LVAT) with CSP than with BiVP (with the best biventricular synchrony being achieved by HBP followed closely by LBBAP).^16^ This has been confirmed *in vivo* by electrocardiographic imaging in patients with bundle branch block, which has additionally shown superior acute haemodynamics with CSP compared to BiVP.^17^

An additional important consideration is that tricuspid valve function is preserved with HBP, as the lead may be positioned on the atrial aspect of the tricuspid annulus or at the commissure between valve leaflets.^18,19^ In contrast, BiVP may result in tricuspid regurgitation due to mechanical interference of the RV lead with normal valve motion.

### Similar or superior outcome with conduction system pacing compared to biventricular pacing

There is an abundance of observational data which indicate that patients with CSP have better clinical outcome than with BiVP,^20–23^ including those with LBBB in whom automatic optimization algorithms were used.^24^ However, the BiVP groups in some of these trials showed a slightly lower response rate than in previous trials. In a meta-analysis of seven randomized trials, CSP was shown to result in significantly greater improvement in LVEF and NYHA class, with a trend in reduced mortality, compared to BiVP.^25^ Although only two of these trials restricted inclusion to patients with LBBB and HFrEF, they both showed either similar^26^ or superior^27^ outcome with CSP. This specific population was studied in the recent CSP-SYNC study,^28^ which also showed non-inferiority of CSP. Large ongoing randomized trials such as Left vs Left (NCT05650658) are currently underway and will contribute to the growing evidence comparing the two treatment modalities.

### Less arrhythmic risk with conduction system pacing

Data from the I-CLAS study indicated that among 1414 propensity score-matched patients, sustained ventricular arrhythmias were very significantly lower in patients with CSP compared to BiVP (HR 0.46; 95% CI 0.29–0.74; *P* < 0.001).^29^ The ventricular pro-arrhythmic effect of BiVP may be explained by its non-physiological electrical activation, which has been shown to result in prolonged QTc and increased transmural dispersion of repolarization, resulting in risk of torsades de pointes and polymorphic ventricular arrhythmia.^30^ Furthermore, colliding wavefronts at the proximity of scar may increase the likelihood of re-entrant ventricular arrhythmias.

The I-CLAS study also showed that new-onset atrial fibrillation (AF) was significantly more frequent with BiVP compared to CSP.^29^ This is most likely due to the modest impact of BiVP on incidence of AF, as shown in the CARE-HF study, where after a follow-up of 29 months, AF was documented in 16% of patients undergoing CRT compared with 14% who received medical therapy only.^31^

### Fewer complications with conduction system pacing

Overall, CSP implantation is a safe procedure.^32–34^ In the large MELOS registry, the most frequently reported complication was peri-procedural perforation of the interventricular septum, occurring in ∼4% of patients.^35^ This event is generally without consequence if promptly recognized and managed by lead repositioning. The most common complication specific to LBBAP is septal venous channel perforation, reported in up to 13% of patients.^36^ This finding, however, has not been associated with adverse clinical outcomes and is likely under-recognized, as contrast injection after lead positioning is seldom performed routinely.

Several independent series have reported a higher overall complication rate with BiVP than with LBBAP, largely driven by coronary sinus lead dislodgment or failure.^21,37^ Biventricular pacing is also associated with phrenic nerve capture, which may occur unpredictably, particularly in super-responders due to ventricular remodelling.^38^ In such cases, lead revision may be required if reprogramming is unable to solve the issue.

### Smaller and simpler systems with conduction system pacing

Conduction system pacing devices that do not require a RV backup or implantable cardioverter defibrillator (ICD) lead *de facto* require one less lead than BiVP systems, thereby reducing intravascular hardware and the larger device pockets associated with bulkier generators and multiple leads. It has been shown that standard ICD leads may also be used to deliver LBBAP,^39^ eliminating the need for an additional pacing lead and further simplifying the system. Recently, lumenless ICD leads have been introduced,^40^ which appear particularly well suited for LBBAP. In pacemaker patients with AF, a CRT pacemaker (CRT-P) generator is required with BiVP, whereas CSP systems only require a single-chamber device. In patients who are in sinus rhythm, single-lead CRT defibrillator (CRT-D) systems delivering LBBAP via a VDD ICD lead may represent a substantially simpler alternative to conventional three-lead CRT-D configurations.^41^

### Cost saving with conduction system pacing

In addition to requiring simpler generators and fewer leads, there are additional factors that contribute to cost savings with CSP compared to BiVP. It has been well established in a large series of 1778 patients that coronary sinus lead thresholds are considerably higher than those of LBBAP leads (1.3 ± 0.7 V vs. 0.7 ± 0.3 V, *P* < 0.001) with significantly more increases in capture thresholds by ≥1 V (7.3% vs. 1.6%, *P* < 0.001).^21^ Coronary sinus leads may show considerable daily fluctuations in capture thresholds, especially if these are high,^42^ meaning that even with automatic capture algorithms, a sufficient safety margin needs to be programmed. Biventricular pacing also results in increased current drain due to delivery of pacing from two ventricular leads. These factors result in reduced battery longevity with BiVP and increased cost (and complications) resulting from additional generator replacements.

In patients implanted with a pacemaker or DF-1 ICD who require upgrade to CRT, the same generator may be used (instead of replacing it with a new CRT device) if remaining longevity is deemed to justify this strategy, which results in cost savings.^43,44^ The LBBAP lead may be connected to the RV IS-1 port of the generator, with capping of the IS-1 pin of the ICD lead. Downgrading CRT-D to CRT-P is also simpler with CSP, as the ICD lead may simply be abandoned in the case of a DF-4 system (whereas only DF-1 systems allow downgrade of CRT-D without implanting an additional ventricular pacing lead).^43,45^

The 2025 European Heart Rhythm Association (EHRA) consensus document on CSP indications advises that CSP indeed ‘may be appropriate to do’ in lieu of BiVP in patients with LBBB and LVEF ≤ 35%. This advice should however be followed by striving to obtain left bundle branch or fascicular capture (as opposed to pure myocardial LV septal pacing),^14,46^ which has been shown to yield better clinical outcome in HF.^47,48^ Proper training for acquiring the necessary skills for CSP implantation is therefore mandatory, as acknowledged by the introduction of this procedure in the EHRA core curriculum.^49^

## Contra

https://orcid.org/0000-0002-4399-5568LeclercqChristophehttps://orcid.org/0000-0001-7529-8735BeharNathalieDeharoJean Claude

Cardiac resynchronization therapy is performed in most cases using BiVP with a lead inserted into the coronary sinus in a lateral or posterolateral vein providing the stimulation of the LV from the epicardium. A RV pacing or defibrillator lead is implanted, either at the apex or at the septum. In case of sinus rhythm, an atrial lead is implanted to provide atrio-ventricular resynchronization. By pacing the RV and LV simultaneously or in a sequential way (RV first or LV first), the resynchronization between the two ventricles and within the LV is achieved. Electrical resynchronization provides a narrower QRS complex, and mechanical resynchronization can be demonstrated using imaging techniques such as echocardiography or cardiac resonance imaging techniques.

Since 1994, the year of the first implantation of a biventricular pacemaker, major technical advances in the technology have been performed (dedicated catheters for the coronary sinus, development of specific leads and recently quadripolar leads, specific algorithms for sequential BiVP, and optimization of atrio-ventricular timing or multipoint pacing).^1^ Importantly, BiVP is available with pacemakers or ICDs.

From the 2000s, many randomized clinical trials have been completed including in total >20 000 patients with reduced LVEF, HF symptoms, and wide QRS and importantly receiving optimal medical treatment. The results of these trials did show that BiVP significantly improved symptoms, quality of life, and functional capacity and may also induce a LV remodelling with a decrease in LV end-systolic and end-diastolic volumes and an increase in LVEF and also mitral regurgitation.^2^ Beyond medical HF pharmacological treatment, BiVP also has a major impact on survival and on HF hospitalizations (*Figure 3*).^3–10^

Based on the results of these randomized trials, the scientific guidelines have included CRT as a major treatment in selected patients since 2005, 20 years ago. The level of recommendation and the class of evidence are different according to the QRS duration and the type of ventricular conduction disorders. In patients with LBBB and wide QRS of 150 ms or more, LVEF not >35% and optimal medical treatment, both 2021 ESC guidelines for cardiac pacing and for HF management recommend CRT with a class I recommendation and a level of evidence A (the highest) to improve symptoms and reduce morbidity and mortality. In patients with QRS between 130 and 149 ms, the level of evidence is IIa, still high. Interestingly, in these two guidelines, there was no recommendation for LBBAP as there was no strong data at the time of the writing of these guidelines or even no data at all. Only HBP was considered an alternative to BiVP in case of failure of LV lead implantation in the coronary sinus.^11,12^

We must acknowledge that BiVP may have some limitations.

First, in ∼5% of cases due to anatomical constraints, it is impossible to place the LV lead in a clinically useful position in the coronary sinus. Pacing thresholds may be high with no pacing of the LV, compromising the longevity of the battery. Phrenic nerve stimulation was at the beginning of CRT a side effect due to the vicinity of the phrenic nerve and the lateral wall of the LV; however, quadripolar leads solve this issue in most cases today. Finally, the quality of the resynchronization may be sub-optimal with the fusion of RV and LV pacing and sometimes intrinsic conduction. Another important factor to consider is that in patients with sinus rhythm, three leads have to be implanted, and we know the relationship between the number of leads and the complications, in particular infections.^13,14^

A drawback of CRT always mentioned in articles dedicated to CRT is the non-responder rate. The non-response to CRT is classically defined in a dichotomous way based on clinical or echocardiographic criteria and is estimated to 30–40%. This number is based on historical studies, but the more recent studies of minimizing the RV pacing indicate that in patients with LBBB and HF, the rate of non-response is rather 20%.^15,16^ A recent consensus paper written by the EHRA and the Heart Failure Association suggested leaving the dichotomous classification of responders and non-responders for a more modern approach based on the terminology used for oncology using ‘remission’ and ‘non-progression’, which looks more realistic for chronic HF.^17^

Physiological pacing may also be achieved by direct pacing of the natural conduction system. Since 2000, HBP has been extensively studied in patients with normal and wide QRS. Theoretically, this pacing modality is very attractive for patients with LBBB without too distal conduction disorders. Unfortunately, the high success rate of implantation is counterbalanced by poor chronic electrical parameters, and this technique is no longer used in patients with LBBB to provide CRT.^18,19^

Another technique to ensure CRT has been developed more recently, the so-called LBBAP that created a craze among the pacing community. From a theoretical point of view, this technique, if well performed with only one lead, may provide a restoration of the natural conduction activation with a more physiological process than BiVP.^20^

But the reality is not so bright. First, LBBAP includes different types of precise locations of pacing. Left bundle branch area pacing terminology is used for the pacing of the left bundle branch itself, pacing of the anterior or posterior left bundle fascicles, and pacing deep in the LV septum. The MELOS registry containing observational data reported by experts of LBBAP (which probably does not reflect the real life with less skilled implanters) did show that in patients with HF and wide QRS, the implant success rate was only 80% and lower than in patients with bradycardia indications.^21^ Conduction system pacing technology, however, remains under development, particularly regarding lead design and device connectivity. The development of new specifically designed catheters with different shapes as well as specific leads should improve the success rate, but this has to be proved. The issue of LBBAP is that from an anatomical point of view, it looks simple; however, we have to be sure that real LBBAP is achieved. A group of worldwide experts led by Haran Burri provides an elegant but yet quite complex algorithm to ensure LBBAP.^22^ The question today is which proportion of LBBAP implanters check very carefully the real delivery of LBBAP.

As with all pacing modalities, CSP comes with complications. Besides the classical complications related to any pacing lead implantation, we have more specific complications such as septal perforation, acute coronary syndrome, and septal haematoma, but we must acknowledge that the complication rate seems to be low and acceptable as illustrated in the MELOS study and further registries.^21^

What is today the clinical evidence of superiority or at least non-inferiority of LBBAP vs. BiVP? To answer this crucial question, we have data from large registries, meta-analyses, and small- to modest-sized clinical randomized trials.

The international LBBAP collaborative group evaluated the results of LBBAP in a multicentre retrospective cohort study of 325 patients with CRT indications. Left bundle branch area pacing was successful in 85%. Left bundle branch area pacing was associated with a significant improvement in NYHA class, a significant decrease in QRS duration, a significant LV remodelling, and finally an improvement in LVEF. Patients with LBBB and/or non-ischaemic cardiomyopathy had significantly greater reductions in QRS duration and improved LVEF compared with patients with non-LBBB and/or ischaemic cardiomyopathy.^23^

Another large retrospective, multicentre, observational, case-control study aimed to evaluate in the real world differences in the clinical outcomes on LBBAP vs. BiVP in 1778 patients (797 patients and 981 patients, respectively) including 1073 patients with LBBB. The primary outcome was a combined endpoint of time to death from any cause or the first hospitalization for HF. Procedural complications occurred in 7.5% in the BiVP group and 3.8% in the LBBAP group (*P* < 0.001).^24^

The primary outcome occurred in 28% of patients in the BiVP group and 21% in the LBBAP group (HR 1.495; 95% CI 1.213–1.842; *P* < 0.001) mainly driven by the HF hospitalization (19% vs. 12%), the mortality being not significant (17% vs. 12%). Left bundle branch area pacing was associated with a significant decrease in QRS duration as well as a significant increase in change in LVEF as compared to BiVP. In LBBB patients, the primary endpoint was significantly lower in the LBBAP group (16.6%) as compared to BiVP (25.7%) related to HF hospitalization but not to mortality. However and importantly, we have to acknowledge that these encouraging results in favour of LBBAP are based on a retrospective registry and not on a randomized prospective clinical trial.^24^

Different meta-analyses have been published comparing BiVP and LBBAP.^25–28^ As an example, the meta-analysis published by Yousaf *et al*.^28^ included 12 studies and 1242 LBBAP patients and 1762 BiVP patients and concluded that LBBAP was superior for electrical and clinical outcomes, but the limitation was the small size of most of the studies.

Randomized clinical trials have been published with the limitation of small-sized trials with no power to compare hard clinical endpoints and with very short follow-up of in most cases 6 months.

Wang *et al.*^29^ published a randomized clinical trial comparing in a 1:1 ratio LBBAP and BiVP in 40 patients with non-ischaemic cardiomyopathy, LBBB, and reduced LVEF. The primary endpoint was the improvement in LVEF. Intention-to-treat analysis showed significantly higher LVEF improvement at 6 months after LBBAP than BiVP (mean difference: 5.6%; 95% CI 0.3–10.9; *P* = 0.039). Left bundle branch area pacing also appeared to have greater reductions in LV end-systolic volume (LVESV) (24.97 mL; 95% CI 49.58 to 0.36 mL) and N-terminal prohormone of brain natriuretic peptide (NT-proBNP) (1071.80 pg/mL; 95% CI 2099.40 to 44.20 pg/mL), as well as comparable changes in NYHA functional class, 6 min walk distance, QRS duration, and rates of CRT response, compared with BiVP. The results of this small trial have to be interpreted with caution especially due to the fact of a high rate of crossover.

The CSP-SYNC trial is a randomized single-centre study testing the hypothesis that LBBAP is non-inferior to BiVP patients in 62 patients with CRT indication and LBBB. The primary endpoint was the change of LVEF at 6 months. Selective LBBA pacing was achieved in 79% of patients. Similar improvement of LVEF was observed in both groups: 14% (95% CI 11.2–16.8) in LBBAP compared to 8.5% (95% CI 5.6–11.2) in BiVP with a mean group difference of 5.6% (95% CI 1.6–9.5; *P* < 0.01 for non-inferiority; superiority was not significant). Same findings were observed for the decrease in LVESV, 6 min walk distance, and NYHA class. Limitations were the small size of the trial conducted in a single centre and the short follow-up time.^30^

The LVET-AT trial compared CSP (mainly LBBAP) and BiVP in patients with CRT indication (35 patients in each group). The primary endpoint was the change in LVAT measured using electrocardiographic imaging. Secondary endpoints were LV reverse remodelling and the combined endpoint of HF hospitalization or death at 6-month follow-up. Crossover was observed in 23% in the CSP arm and 6% in the BiVP arm. A similar decrease in LVAT was observed in both groups (−26 + 26 ms for CSP vs. −21 + 20 ms for BiVP; *P* < 0.01 for non-inferiority). Similar changes in LVESV or similar rates of mortality or HF hospitalization were observed with significant *P*-values for non-inferiority. The small size of the population and the short follow-up are major limitations of this study.^31^

Interestingly, the PHYSIO-SYNC-HF trial including 173 patients in a 1:1 ratio, presented at the ESC congress, did show that CSP was associated with an increase in death or HF hospitalizations as compared to BiVP (HR 2.35; 95% CI 0.99–5.61) as well as a lower increase in LVEF. As the results of this study have not been published yet in a manuscript form, interpretation of the results has to be done very cautiously. However, this is so far the largest RCT comparing CSP with BiVP for HF. Only the design of the study has been published.^32^

All these trials have tested LBBAP using cardiac pacemakers as the cardiac implantable electronic devices because of the lack of available ICD leads dedicated to LBBAP. This is a limitation as in most of cases CRT is provided with CRT defibrillators. For CSP, an additional high-voltage lead is necessary, further increasing the number of leads crossing the tricuspid valve. A conventional ICD lead has been tested successfully in terms of defibrillation thresholds and electrical parameters.^33^ Specific ICD leads dedicated to LBBAP are under clinical evaluation.

In 2023, the Heart Rhythm Society, the Asia Pacific Heart Rhythm Society, and the Latin American Heart Rhythm Society published guidelines on physiological cardiac pacing. For patients with HFrEF and LBBB with a QRS duration of >150 ms, BiVP has a class I recommendation and LBBAP a class IIb, clearly reflecting the difference in strength of evidence (*Table 1*) supporting the two pacing modes.^34^

An ESC-EHRA consensus on CSP was published this year. We must remind that this document is not an ESC guideline document that can provide recommendations but a document providing advices. For patients with LVEF < 35% and LBBB, BiVP was ‘advice to do’ and CSP was ‘may be appropriate to do’, so a clear difference between the two pacing modalities is apparent.^20^

Finally, lead extractability is an evolving concern with CSP. Because the leads are implanted deeply within the septal myocardium or in the His region, fibrotic encapsulation can occur, potentially making extraction more challenging than with conventional RV or coronary sinus leads. Current evidence remains limited and short-term, and additional long-term studies are required to evaluate the safety and extraction success rates relative to CRT leads, which benefit from a substantially larger and longer clinical experience.^35–38^

Thus, LBBAP seems to be a very attractive technique with a high rate of success but with the limitations that implanters provide real LBBAP. Previous results of registries, meta-analyses, and small randomized trials are encouraging but do not support the replacement of BiVP by LBBAP with strong evidence. Large RCTs are currently recruiting and will probably clarify the situation. Careful selection of patients for LBBAP needs to be better clarified as we know that a substantial number of patients have distal conduction disease, which cannot be corrected by LBBAP. In such cases, BiVP and CSP can be synergistic as shown in the LOT study.^39^
